# Exploring the Role of Conscious and Unconscious Processes in Hypnosis: A Theoretical Review

**DOI:** 10.3390/brainsci14040374

**Published:** 2024-04-12

**Authors:** Gavriel Knafo, Joel Weinberger

**Affiliations:** Derner School of Psychology, Adelphi University, Garden City, NY 11530, USA; gavrielknafo@mail.adelphi.edu

**Keywords:** hypnosis, unconscious processes, conscious processes, neurocognitive models, attribution theory, socio-cognitive model, Mesmer, Freud, unconscious gatekeeper

## Abstract

This review provided a comprehensive examination of various theories that attempt to explain hypnosis, focusing on the interplay between conscious and unconscious processes. We conducted a thorough analysis of key theories, from historical origins to recent models centered on cognition, social factors, and attributions. A central theme emerged: the critical role of the unconscious as a “gatekeeper” that modulates and guides the hypnotic experience. This notion appears in various forms across many theories, with the unconscious actively shaping and regulating the flow of information between conscious and unconscious realms during hypnosis. Understanding this dynamic interplay is crucial for comprehending the complex nature of hypnosis. The synthesized view of the unconscious as a “gatekeeper” offers a framework for integrating insights from diverse perspectives and highlights the centrality of unconscious processes in shaping hypnotic phenomena. Future research should further investigate the mechanisms of this unconscious “gatekeeper” role and its impact on hypnosis.

## 1. Introduction

Hypnosis is a fascinating phenomenon that has captivated the minds of theorists, practitioners, and the general public for centuries. It is a process involving focused attention, reduced peripheral awareness, and enhanced capacity to respond to suggestion [1]. The hypnotic experience often involves alterations in perception, sensation, emotion, thought, or behavior [2]. Despite its long history and widespread interest, the exact mechanisms underlying hypnosis remain a subject of ongoing scientific inquiry and theoretical debate.

When we see or hear the word hypnosis, we are instantly transported to a seemingly magical world in which one person gains control over the mind and behavior of another. Indeed, hypnosis has long been regarded as a mysterious activity that has succeeded in captivating the minds of theorists, practitioners, and the general population. French psychiatrist, Jean Martin Charcot, who worked at Paris’s prestigious Salpêtrière Hospital in the late 1800s, became well known for his Tuesday sessions during which he hypnotized his (mostly female) patients before an audience of men of science, including Sigmund Freud, as well as many of Paris’s VIPs: artists, actors, writers, and aristocrats [3]. The spectacle showcased women and girls diagnosed with hysteria, the malady of that era. Charcot paraded them on stage, one after the other, commanding them to either produce an act of hysteria or turn off the symptoms they complained of. A woman who believed she was paralyzed could suddenly walk. Another who believed she was blind could suddenly see. The audience was shocked, titillated, and “entranced” themselves. Previously accused of malingering, these cases seemed to reveal two profound truths: (1) that there exist two states of consciousness, one unaware of the other at any given time, and (2) that one can acquire the skill to influence a person to enter said state of consciousness.

People began speaking about a condition seconde, a second consciousness—what we might now call the unconscious. This paper is a theoretical review that embarks on a journey through a landscape of various theories that have tried to make sense of the phenomenon of hypnosis by attempting to unravel some of its complexities and apparent contradictions. Studies and theories were selected based on their relevance to elucidating the relationship between conscious and unconscious mechanisms in hypnosis, with a focus on seminal works that shaped the field’s understanding of this topic. We emphasize and critically analyze the role each theory places on unconscious and conscious processes, as well as the interplay between the two.

We begin with the discovery of hypnosis by Franz Mesmer and his followers, who were the first scientifically minded Western thinkers and practitioners to discover and try to explain this perplexing phenomenon. We then transition to Freud’s psychoanalytic theory, which offered insights into the role of unconscious processes revealed by the hypnotic experience, followed by theories that debate whether hypnosis represents a pathological or a normative phenomenon. Our exploration extends to neurocognitive theories, providing a modern lens through which we can view the intricate workings of the brain/mind during hypnosis. Next, we shift our focus to theories centered on conscious cognition, social factors, and the interplay of conscious and unconscious attributions. Throughout these discussions, a central theme emerges: the indispensable role of unconscious processes as a “gatekeeper” that modulates and guides the hypnotic experience, a notion that appears in many of the theories, even though some do not state it explicitly.

## 2. Origins

German physician Franz Mesmer was enamored by a theory proposed by Richard Mead claiming that the gravitational pull from planets affects human health via an invisible fluid that acts according to the laws of magnetism [4]. When this fluid flowed freely, people functioned well, but when it did not, they experienced emotional and physical problems. Mesmer believed that if someone was skilled and gifted with their own powerful “animal magnetism”, they could affect these blocks. This healer could help the sufferer to enter a special state during which their fluid could be influenced to flow freely once again, thereby restoring harmony. Mesmerism, or magnetism, as Mesmer termed it, was based on a physical model of fluid adjustment. Although his model did not explicitly refer to the conscious or unconscious mind, it did imply that the hypnotist can exert conscious control over their own magnetic fluid, whereas the hypnotized person cannot do so. It did not take long for someone to take the next step and explicitly refer to conscious and unconscious processes.

Puységur (1751–1825), a disciple of Mesmer, shifted the discourse from a quasi-mystical physical force to a psychological phenomenon [5]. He introduced the concept of “somnambulism”, a sleep-like state during which the individual purportedly gains access to his or her unconscious mind. This state, he claimed, is reached by establishing “rapport” between the hypnotist and the subject, opening up an access point for an altered state of consciousness which is entered without the subject’s conscious control. Puységur was among the first to explicitly introduce the idea that unconscious processes could be accessed and manipulated during hypnosis and, perhaps more importantly, that the therapist–patient relationship is critical to this process [6]. 

Hypnotism then entered a phase wherein it was considered controversial by the scientific community. It did not return to respectability until the late 19th and early 20th centuries [7]. At that time, theorists and practitioners like Charcot, Liébault, and Bernheim sought to ground hypnosis in empirical observation and scientific rigor, allotting it some degree of scientific respect. Their work laid the foundation for later theories that have continued to grapple with the role of conscious and unconscious processes in hypnosis [6,8,9].

## 3. Sigmund Freud

In 1885, Freud traveled to Paris, where he stayed for five months to study under Charcot [10]. While there, Freud learned about hypnosis and the psychogenesis of mental conditions like hysteria. Early in his career, Freud applied hypnosis to his hysteric patients, primarily as a tool to access repressed memories stored in what he understood as the unconscious mind [11]. Freud’s hypothesis, although not directly articulated, implied that during hypnosis, suggestion helps the unconscious mind to release previously inaccessible information so that it can become conscious. This suggests an active mechanism within the unconscious that regulates information flow to the conscious mind, akin to a gatekeeper. This part of the unconscious selectively determines which information is transmitted and which remains hidden, thereby playing a critical role in the process of hypnosis. Freud [11] eventually abandoned hypnosis, which he said did not work well. Freud argued that although hypnosis bypasses one’s resistance, it can result only in temporary relief and symptom substitution. He replaced it with his method of free association, i.e., for the patient to say whatever comes to mind no matter how irrelevant or distasteful it might be. He believed that this method worked better than hypnosis and led to clues about what a patient repressed or warded off. Having a patient lie on the couch with minimal stimulation (not seeing the analyst’s facial expressions and body language) enhanced the effects of free association by relaxing the patient’s defensiveness and opening the door to previously inaccessible material. The famous analytic couch was thereby born.

Free association, Freud found, could achieve similar results to hypnosis while also dealing with a patient’s resistances. Resistance, he claimed, was not simply a hindrance to treatment, but a clue to what was being repressed. One of Freud’s lasting contributions to our understanding of unconscious processes and therefore also of hypnosis involved his positing that the unconscious mind was not simply a passive reservoir but an active participant in shaping behavior and experience, hence the term “dynamic unconscious”. He also theorized that there exist two types of the unconscious. Her termed them the system unconscious and the preconscious system. The preconscious was simply what was not conscious at the moment but could become conscious easily. The unconscious was defended against and had a much more difficult time achieving consciousness, hence the need for free association and minimizing outside stimulation. Although hypnosis could also access unconscious processes, it could not do so as well as the free associative method.

## 4. Pathology versus Normality

The theoretical landscape of hypnosis has been marked by divergent perspectives on the role of consciousness since the contrasting views of Jean-Martin Charcot and the team of Ambroise-Auguste Liébault and Hippolyte Bernheim [7]. In 1882, Charcot posited that hypnosis is a pathological state akin to hysteria, characterized by an altered, but not entirely absent, consciousness, suggesting a neurological dysfunction [7]. Pierre Janet, too, believed that the effects of hypnosis are similar to the pathological dissociation observed in hysteria [12]. In fact, he averred, as did Charcot, that only hysterics could be hypnotized [7,12]. The views of Charcot and Janet differed from Freud’s non-pathologizing view of hysteria and hypnosis. Like Freud, Liébault’s (1866) and Bernheim’s (1886) suggestion-based models also viewed hypnosis as a normative psychological phenomenon, emphasizing the mind’s responsiveness to suggestions, as opposed to considering it as diagnostic of mental illness [7,12]. These differing viewpoints continue to be instantiated in the debate over hypnosis as either a pathological condition or a normal state of enhanced suggestibility [13].

These models offer a range of perspectives on the role of consciousness in hypnosis, each with its own set of implications and limitations. Charcot’s model, although historically significant, has been largely discredited, partly due to its pathological framing [7]. In addition, Janet (who also believed that hypnosis was related to pathology) identified methodological problems with it that discredited Charcot’s model of the stages of hypnosis and hysteria [12]. Liébault’s and Bernheim’s suggestion-based models have fared somewhat better, facing challenges mainly when attempting to explain phenomena such as post-hypnotic amnesia [14].

## 5. Dissociation and Neo-Dissociation

Like Janet before him, Hilgard’s “Dissociated Control Theory” (1992) posits that hypnosis involves a division of consciousness into different streams [15]; yet, it differs from Janet’s conceptualization in two ways. First, hypnosis is not seen as indicative of pathology. Second, Hilgard posits a different type of dissociation than Janet. For Hilgard, one stream obeys the hypnotist whereas the other remains a “hidden observer.” This theory suggests that during hypnosis, the mind can actively partition itself, allowing for separate control of conscious and unconscious processes. This aligns with Kihlstrom’s (1987) arguments for the existence and importance of unconscious cognitive processes that influence perception, memory, and action outside of conscious awareness [16]. He later (2013) argues that hypnosis involves unconscious processes [17]. The partitioning process in Hilgard’s theory is necessarily modulated by an unconscious governor which is crucial for managing the distinct streams of consciousness. Without such a governor, the mind would struggle to maintain separate conscious and hidden observer states during hypnosis, leading to a potential collapse or blending of these states. This unconscious regulator ensures that while one part of the mind responds to the hypnotist, the other part, the ”hidden observer”, remains detached yet aware, providing a necessary balance and oversight to the divided consciousness. Hilgard highlights the idea that one can intensely focus on certain tasks or thoughts during hypnosis while the “hidden observer” remains detached and aware, but not participating. Given that it does not participate, the “hidden observer” represents a secondary unconscious process separate from the role of the “gatekeeper”. 

A similar notion, grounded in the neurocognitive model of brain modularity, is promulgated by the cognitive neuroscientist Michael Gazzaniga (1998). Gazzaniga believes that part of the brain (a brain module) observes what we are doing and constructs a narrative about it [18]. This “interpreter” complements Hilgard’s concept of the hidden observer, and it aligns with our notion of the unconscious “gatekeeper”. It usually makes sense of our actions and thoughts correctly, but sometimes it creates narratives that do not align with reality. Both Hilgard’s and Gazzaniga’s theories explore the multifaceted nature of consciousness, each highlighting different aspects of how our minds perceive, processes, and narrate our experiences and states of consciousness [15,18]. Bob (2014) similarly references the role of the unconscious in modulating or “gating” the contents of the conscious experience during dissociative states like hypnosis [19]. Although neither Hilgard nor Gazzaniga posit a theory of hypnosis that comprehensively models the mechanisms of the hypnotic experience, they agree on the modularity of the mind. That is, they both emphasize an unconscious observer and interpreter, respectively, as well as a conscious part of the brain that is receptive to a subjectively sensible narrative. This seems to address the following question: “how does the subject make sense of the experience?” Yet it does not address the included scope of the hypnotic experience and its functions other than the fact *that* it functions. 

Kenneth Bowers’ (1990) neodissociation theory takes Hilgard’s dissociated control theory a step further by explicitly focusing on unconscious processes [20]. Unlike Hilgard, Bowers posits that the unconscious itself can be segmented, and that these segments can be independently activated or deactivated during hypnosis. This adds a layer of complexity to our understanding of the unconscious, suggesting that it is not a monolithic entity but a composite of different processes that can be individually targeted and engaged. Bowers’ theory sheds light on the unusual behavior often observed in hypnosis sessions by suggesting that it results from the unique manipulation of unconscious processes. In this model, specific mental processes, usually fundamental to our regular functioning, are selectively activated or deactivated, leading to behavior that deviates significantly from the norm. Integrating this theory of a sort of atomized unconscious with Freud’s findings on hypnosis and its ability to access repressed memories evokes questions about whether one unconscious process is an unconscious gatekeeper for other unconscious processes and whether there is yet another unconscious process that acts as a central controlling unit of the various other unconscious processes. If so, is the functionality of this unit transferrable to an external source (the hypnotist) who acts as an alternate power source to this conductor of other unconscious processes?

Bowers’ theory, with its emphasis on the modularity and selective control of various unconscious processes, leads us to ponder the extent of influence a hypnotist might exert over these unconscious segments. This question segues into the development of cold control theory as explicated by Dienes and Perner [21]. Cold control theory, like Bowers’ neodissociation theory, has similarities to Hilgard’s dissociation control theory. Cold control theory postulates a distinct bifurcation of consciousness that takes place during hypnosis, effectively separating hypnotic suggestions from the individual’s conscious awareness. According to cold control theory, during hypnosis, there is a “cooling” or reduction in conscious control over one’s thoughts and actions because individuals intentionally allow for their actions and thoughts to be guided by external suggestions without the usual conscious scrutiny or decision-making process that might otherwise prevail. This “cooling” occurs as hypnotic subjects adopt an attitude of dispassionate engagement, wherein they are aware of their actions but do not engage with them in a typical, self-reflective manner. Essentially, the person experiences detachment from their own decision-making process, allowing for hypnotic suggestions to direct their thoughts and actions without the usual conscious oversight. In the hypnotic state, subjects often experience their responses to hypnotic suggestions as if they are occurring independently of their conscious will. Cold control theory therefore claims that individuals attribute their hypnotic behaviors to the hypnotist’s commands rather than to their own free will. 

Although cold control theory is presented as a hypnotic model that relies on consciousness, the model suggests a sort of gray area between conscious and unconscious mechanisms at play in the “cooling” process. On the one hand, Dienes and Perner’s view that individuals consciously relinquish control over their actions is plausible because many individuals expect that hypnosis will generate an altered state of mind. On the other hand, cold control theory can also align with the notion of an unconscious gatekeeper that restricts or allows for the flow of information from unconscious to conscious processes. In this case, what might normally be encompassed in the conscious portions of the brain is relegated to unconscious systems via the processing of hypnotic cues. The mechanism is necessarily mediated by unconscious processes, as individuals do not choose whether to be consciously aware of information; it occurs outside of their awareness.

Palfi et al. (2021) tested the cold control theory’s prediction that hypnotic responses are driven by unconscious intentions without accurate higher-order thoughts (HOTs) of intending [22]. Using posthypnotic suggestion, they elicited motor responses in high-hypnotizable subjects that lacked feelings of voluntariness. The results support the idea that unconscious volitional control can occur in hypnosis, independent of conscious intentions. Similarly, Schlegel (2015) used posthypnotic suggestion to elicit volitional movements without accompanying sensations of will in high-hypnotizable subjects [23]. They found that readiness potentials (RPs) still preceded these "non-conscious volitional" movements, suggesting the neural processes indexed by RPs can occur independent of conscious intentions, indicating that unconscious processes can independently operate. 

## 6. Neurocognitive Models and Hypnosis

There are basically three major neurocognitive models of the mind [9]. Each has different implications for hypnosis. The “Massive Modularity” model, popularized by Stephen Pinker (2005), posits that rather than being a general-purpose cognitive tool, the human mind is composed of specialized independent units, termed modules, that evolved to handle specific tasks [24]. Weinberger and Stoycheva [9] interpret this model as one that minimizes the role of consciousness in mental functions. Likewise, if the mind consists of specialized, task-specific modules, this inherently reduces the scope for any kind of unconscious meta-control. This is because, in a system of dedicated unitaskers, some sort of overseeing unconscious would only have limited capacity to regulate or influence these independent modules. In fact, it is highly unlikely that such an overseer would exist at all. Consequently, within the framework of this model, one would almost have to argue that no one conscious or unconscious process plays a significant role in guiding hypnotic processes. The modular structure of the mind does not seem to allow for such overarching control or facilitation.

The “Connectionist” model, particularly Parallel Distributed Processing [25], offers another way of conceptualizing the mind and therefore hypnosis. In this model, in contrast to massive modularity, it is theorized that networks of simple, neuron-like units, connected to each other and distributed throughout the brain, communicate and send/receive signals. Moreover, multiple units can (and do) operate at the same time (in parallel), with information stored in multiple places (distributed) but connected to one another rather than being located in a single part of the brain. In other words, this model posits that cognitive processes are distributed across a network of interconnected nodes in the brain that operate largely unconsciously. By arguing that cognitive processes are distributed in this way, the model suggests that the mind and brain operate almost entirely unconsciously, as we do not have the conscious ability to discern the location where information is stored or the area of the brain that is executing a task (especially if it is occurring in multiple places, [9]. This could imply that the hypnotic state is achieved by manipulating these unconscious networks to produce specific behaviors or experiences via some variation in how the network is modulated.

Michael Anderson’s “Neural Reuse” theory describes a flexible, dynamic use of brain areas for various cognitive functions. Essentially, the same neural circuits are used for multiple tasks and purposes [26]. It posits larger units than PDP does, but smaller, more elementary, units than massive modularity [9]. As with PDP, it is challenging to find a place for consciousness in neural reuse [9]. In terms of hypnosis, we can speculate that if neural circuits developed for one purpose but can also be “reused” for other purposes (a central tenet of neural reuse termed, in evolutionary theory, exaptation) then hypnosis may direct or even repurpose circuits that evolved for one purpose to perform others during hypnosis, thereby actively shaping the hypnotic experience. 

## 7. Attribution

Attribution theory, which was initially posited by Fritz Heider [27] and expanded upon by Harold Kelley [28] and Bernard Weiner [29], focuses on how individuals attribute causes to events and behaviors. Although this theory is not traditionally linked to hypnosis, we believe that it nevertheless proposes a useful framework for helping us to understand the hypnotic state by offering an empirical model of the different ways that people interpret their experiences. It can achieve this by applying its analyses to whether the person attributes the hypnotic experience to their own agency, the hypnotist, or to other external forces [8].

Two biases in attribution are particularly relevant to hypnosis: the correspondence bias and the self-serving bias. The correspondence bias, sometimes also known as the fundamental attribution error, was first identified by Jones and Harris [30]. It describes the tendency for people to attribute the behaviors of others to their personality traits while underestimating situational influences. At the same time, they are more likely to attribute their own behavior to the situation over personality characteristics. In hypnosis, this might lead a subject to attribute the effectiveness of hypnosis to the situation they find themselves in, which would include the hypnotist’s skills as well as the hypnotic setting (alone in a room, focusing on an object, etc.). The self-serving bias, outlined by Campbell and Sedikides [31], involves attributing successes to internal factors and failures to external factors. Within the context of hypnosis, a subject might attribute a successful hypnotic session to their own ability to relax or concentrate, while attributing a less successful experience to external distractions or the hypnotist’s ineffective technique. Both of these attributions, though made unconsciously, influence one’s conscious perception of why the hypnosis session was effective/ineffective that may or may not be accurate.

Recent models of attribution theory necessarily focus on unconscious processes, at least in part, as they relate to factors that contribute to an individual attributing events one way or another, often in ways that are not accurate. Although attribution theory does not directly posit it, one could understand attributional phenomena in terms of a governor of unconscious processes that biases conscious cognition. In the case of hypnosis, these attributions can lead to predictable biases that can yield certain tendencies that change how people conceive of their hypnoses. 

Bar-Anan, Wilson, and Hassin’s (2010) model of attribution theory suggests that people form causal explanations based on the accessibility, plausibility, and self-enhancement of information [32]. This means that individuals are more likely to attribute causes to factors that are immediately available to their consciousness, make logical sense to them, and align with their self-view. This model is relevant for understanding phenomena that are said to involve altered states of consciousness, such as hypnosis. Bar-Anan et al.’s framework can provide insights into the ways in which subjects interpret their hypnotic experiences [32]. The suggestions given by the hypnotist and the hypnotic environment itself are highly accessible to the subject. These factors make certain attributions more plausible to the subject than they would be in a non-hypnotic state. For instance, a subject might attribute their ability to perform unusual tasks or experience altered perceptions not to their own volition, but to the hypnotist’s influence or the power of the hypnotic setting. This could lead to a diminished sense of personal agency and an enhanced perception of the hypnotist’s control, which are crucial for the effectiveness of hypnosis. 

In the same vein, Cusimano and Goodwin’s (2019–2020) research into what can be termed the self-other controllability effect reveals how individuals tend to believe that others have more control over their internal states, such as emotions and beliefs, than they themselves do [33,34]. This bias can significantly impact interpersonal dynamics, as it leads to attributing more responsibility and blame to others for their actions while perceiving one’s own actions as less controllable and therefore less blameworthy. Applied to hypnosis, this theory suggests that subjects might view the hypnotist’s role in the hypnotic experience as dominant and under the hypnotist’s control, whereas they might see their own role as passive and influenced by external factors. This perception reinforces the hypnotic dynamic, wherein the hypnotist is seen as the controller and the subject as the one being controlled. 

## 8. Salience and Attribution

“Salience”, as defined by Smith and Miller (1979), refers to noticeable or prominent factors in shaping our attributions, because what is salient is often perceived as causal [35]. This becomes crucial in hypnosis, where the most noticeable elements in a session—be they novel, intense, or emotionally impactful—disproportionately shape the subject’s perception and interpretation of the experience. These salient aspects often overshadow elements that are not consciously noticed. They are more often seen as causal, whereas those aspects that are less noticed are not understood as causal. And the most salient characteristic of the environment in the hypnotic situation is the hypnotist along with their words and behaviors.

For instance, the actions and words of the hypnotist can become highly salient to the subject. If the hypnotist’s tone of voice, choice of words, or even physical presence are perceived as particularly striking or authoritative, these factors can greatly amplify the hypnotic experience. The hypnotic subject might attribute either a deeper state of relaxation or a heightened responsiveness to these salient features of the hypnotist. Certain stimuli would therefore stand out without the subject’s explicit awareness. These could include cues from the hypnotist, such as specific gestures or the pacing of speech, which might not be consciously noted but nonetheless influence the subject’s state of mind and subsequent attribution. Unconscious salience can guide the subject’s responses to suggestions and shape their overall experience in ways that may not be immediately apparent to them. These cues, although impactful, are not retained in the subjects’ post hoc explanatory narrative.

The “Attribution-Value Model” of Martinko and Mackey (2019) extends attribution theory to emphasize the significance individuals assign to different attributions [36]. This aligns with Bar-Anan, Wilson, and Hassin’s perspective on the role of accessibility [32], plausibility, and self-enhancement in shaping causal explanations. This integration suggests that the effectiveness of hypnotic suggestions is influenced not only by their source but also by the subject’s value system and unconscious biases towards self-enhancement. A subject highly valuing the hypnotist’s expertise might find suggestions more effective, whereas one prioritizing personal agency might lean towards self-generated thoughts. This personal orientation, intertwined with the salience of external suggestions or internal thoughts, can shape the hypnotic experience. Salient factors, such as the hypnotist’s actions and words, are unconsciously prioritized, guiding the subject’s responses and shaping their experience in ways that align with their self-view and the immediate, plausible attributions accessible in the hypnotic state. The process of attribution through salience, underscored by unconscious preferences and self-enhancing biases, thus exerts significant control over the direction and impact of hypnosis, making the hypnotic experience a complex interplay of personal values and unconscious salience.

Cultural factors can also possess salience. A model of culturally based attribution, as elucidated by Lynn and Rhue (1988), highlights the profound impact of cultural factors on attribution patterns [37]. This model suggests that the cultural background of an individual can influence the ways they attribute causes to their behaviors and experiences. According to this model, one’s cultural background renders certain elements salient and these then tend to play a critical role in determining how subjects perceive and interpret their hypnotic experiences. In cultures that place a high premium on individualism and personal agency, subjects are likely to attribute their experiences in hypnosis to internal factors such as their mental strength and/or concentration. This internal attribution can lead to a heightened sense of control and, potentially, a more profound hypnotic experience, as subjects believe they are actively contributing to the process. Conversely, in cultures where external forces or collective agency are emphasized, subjects might be inclined to attribute the effects of hypnosis to the hypnotist’s expertise, spiritual influences, or other external factors. Here, the hypnotic experience may be perceived as a more passive one, with the subject viewing themselves as under the influence of external agents. This cultural backdrop, therefore, is not merely a peripheral factor but a central element in shaping the hypnotic experience. It determines the framework within which subjects construct their understanding of experiences like hypnosis, influencing everything from their expectations to their responsiveness to hypnotic suggestions.

## 9. Automatic vs. Controlled Attributions

The adaptive experiential theory of hypnosis proposed by Alldredge and Elkins (2023) differentiates between automatic and consciously controlled attributions, thus providing a nuanced understanding of the ways in which people form conscious and unconscious attributions about their hypnotic experiences [38]. Automatic attributions, characterized by their rapid, intuitive nature, are unconsciously processed judgments about the causes of behaviors or events. These occur instantaneously and without deliberate thought. As it applies to hypnosis, a subject might automatically attribute their feelings of relaxation to the hypnotist’s calming voice, an attribution that significantly influences their immediate response to hypnotic suggestions. Controlled attributions are more deliberate and conscious, involving a reasoned analysis of events and requiring focused, conscious effort. Subjects might engage in this type of attribution during or after hypnosis, thoughtfully considering the reasons behind their responsiveness to certain suggestions. The notion of controlled attribution provides a role for more conscious cognition in the salience ecosystem. The subject, presented by the unusual experience of being under hypnotic influence, wishes to align the experience with one that makes sense, so they seek out salient factors that would explain it. Controlled attribution, when it does take place, offers a ripe opportunity for misattribution as any factors that were unconsciously salient will be missed in the attribution process, leading back to the notion of an unconscious interpreter [18]. 

## 10. The Socio-Cognitive Model

The socio-cognitive model proposed by Nicholas Spanos presents a departure from traditional understandings of hypnosis as an altered state of consciousness, trance state, or interaction of conscious and unconscious processes [39]. Instead, Spanos’s model conceptualizes hypnosis as a fundamentally social interaction between the hypnotist and the subject [39]. In this framework, the hypnotist provides verbal suggestions and guidance, directing their subject’s focus of attention and concentration toward specific experiences, perceptions, behaviors, or tasks. Rather than passively entering a dissociated psychological state, marked by absorption and susceptibility to suggestion, subjects are believed to make a volitional choice to participate in the socially defined role of someone who is being hypnotized. This participation involves consciously and purposefully narrowing one’s awareness and becoming absorbed in the specific mental imagery, ideas, or perceptions being suggested by the hypnotist. Thus, for Spanos, hypnosis represents not an altered state of consciousness, but rather a focused, engaged concentration and willful compliance motivated by the social dynamic and context established in the hypnotic setting. This reimagining of hypnosis as dependent on socio-cognitive interpersonal factors marks a shift from earlier understandings based predominantly on the notion of a special trance state characterized by unconsciousness and automatism.

Although Spanos conceives of hypnosis as largely conscious, key tenets of the socio-cognitive model can nonetheless be understood as emphasizing unconscious processes. Rather than passively entering a distinct “trance state” marked by an altered consciousness, hypnotic responses can be understood as individuals actively, even if unwittingly (unconsciously), modifying their actions to align with normative conceptions and narratives about appropriate conduct under hypnosis. In this sense, the model proposes that hypnotic phenomena arise predominantly from an individual’s unconscious adherence to socially conditioned roles and learned expectations of how one should behave when hypnotized, as opposed to inherently experiencing a split in consciousness. By integrating key tenets of social psychology—particularly role theory along with the impact of sociocultural narratives and situational demands—the socio-cognitive model enriches the conceptual toolkit by arguing that hypnotic behavior, at least in part, is a product of dynamic interactions between social contexts, unconscious processes, and conscious efforts to play into one’s cultural role.

The socio-cognitive model may be said to integrate attribution theory principles, enriched by insights from Bar-Anan, Wilson, and Hassin [32]. This model elucidates the complex interplay between an individual’s attributions of causes to behaviors and the potent influences of social cues, contextual factors, and personal beliefs within the hypnotic environment. It aligns with Bar-Anan, Wilson, and Hassin’s [32] emphasis on accessibility, plausibility, and self-enhancement, suggesting that attributions during hypnosis are significantly shaped by factors that are immediately perceptible, logically coherent, and self-affirming. The socio-cognitive Model extends beyond the mere identification of involuntary experiences and salience, incorporating the profound impact of sociocultural narratives and personal ideologies on the hypnotic experience. This framework posits that the hypnotic state is not merely a passive reception of suggestions but a dynamic construct where personal biases, social expectations, and the salience of hypnotic cues interact to form a rich tapestry of cognitive attributions. The model thereby offers a comprehensive understanding of hypnotic responses as constructs influenced by a broader spectrum of sociocultural and psychological dynamics, underscoring the complexity of the hypnotic phenomenon.

## 11. Summary: The Unconscious as Gatekeeper

We believe in the importance of emphasizing the critical role of an unconscious “gatekeeper” in the realm of hypnosis, a theme recurrently addressed throughout this paper. The role unconscious processes play as a gatekeeper actively modulates the flow of information and influence between various conscious and unconscious processes. Although many believe the unconscious and conscious parts of our minds are separate, and that each is impermeable to the other, the theories described above demonstrate how much hypnosis helped make clear the degree to which the two are in a constant state of interaction. Nothing is purely conscious or purely unconscious. Moreover, there is no firm line that differentiates the two [9]. Freud (1900) was the first to describe the dynamic between the unconscious and conscious, hence one of the names for his theory is “psychodynamic” [40]. Freud’s conception of the preconscious serves our conception of a gatekeeper well. Not everything from the various unconscious processes passes through this preconscious process; however, in relaxed states, like the one experienced before falling asleep, when lying on a couch, while dreaming, or during hypnosis, the preconscious processes open their gates, allowing freer access to other unconscious processes. Freud [40] eventually propounded the view that consciousness and unconsciousness were qualities of experience rather than separate systems, which is in line with what we are arguing here. Also, see Weinberger and Stoycheva, who come to a similar conclusion based on more recent empirical data and neurocognitive models [9].

Hilgard’s dissociated control theory introduced the concept of an unconscious governor, a critical component in the division of consciousness into distinct streams during hypnosis, further underscoring the regulatory role of unconscious processes [15]. This theory aligns with the adaptive experiential theory of hypnosis [38], which differentiates between automatic and controlled attributions, attributing the former to unconscious judgments that significantly influence immediate responses to hypnotic suggestions.

Cold control theory [21] further illustrates the delicate interplay between conscious and unconscious mechanisms, particularly in the “cooling” process where individuals consciously relinquish control, allowing for unconscious processes to guide their actions and thoughts. In cold control theory, unconscious processes serve as mediators. This is achieved when unconscious processes evaluate and filter experiences based on their relevance and appropriateness for conscious processing. Like Freud’s preconscious, the unconscious “gatekeeper” determines the elements of experience that are admitted to conscious awareness and those that remain unconscious. This selective permeability profoundly influences how individuals process hypnotic cues, thereby shaping their hypnotic experiences.

Moreover, the segmentation and independent activation or deactivation of unconscious processes, highlighted in neodissociation theory [20], further reinforce the notion that what is often called the unconscious is actually a composite of different processes with gatekeeping capabilities rather than a monolithic entity.

Certain neurocognitive theories, particularly connectionism—specifically Parallel Distributed Processing [25]—and neural reuse [26] can be said to position unconscious processes as central players in hypnosis. Connectionism posits that cognitive processes are distributed across networks of interconnected nodes that operate largely unconsciously, suggesting that hypnotic states can be achieved by manipulating these networks to produce specific behaviors and experiences. Similarly, the neural reuse theory, which describes the flexible use of brain areas for various cognitive functions, implies that hypnosis may direct or repurpose neural circuits for tasks they were not originally evolved to perform. Both theories underscore the pivotal role of unconscious processes in modulating and reshaping cognitive processes during hypnosis, enhancing our understanding of how unconscious mechanisms contribute to the hypnotic experience. 

Spanos’ socio-cognitive model [39] also suggests that an individual’s adherence to socially conditioned roles and expectations, witnessed in hypnosis, is predominantly an unconscious phenomenon. This model, along with attribution theory, highlights unconscious influences in determining the salience of certain elements while in hypnosis, guiding a subject’s responses in ways not immediately apparent to their conscious awareness.

In summary, we highlight the role of a gatekeeper as an unconscious process that determines the conscious processes that can or cannot be accessed while in a hypnotic state. This role is evident in the variety of very different theoretical models reviewed here and is helpful for understanding the hypnotic experience. Unconscious processes not only influence what is permitted to enter conscious awareness, but they also actively shape and modulate the hypnotic experience. Last but not least, it is primarily unconscious processes that affect a subject’s interpretation of their behavior while hypnotized. 

## 12. Future Directions and Interplay of Conscious and Unconscious Processes

The focus of this paper is on the subject and state of hypnosis. However, much of what we argue here also has relevance to our understanding of the interrelationship between unconscious and conscious mental processes more generally. In fact, one could say that theories of hypnosis are essentially theories of the interaction between conscious and unconscious processes. This is a subject that is only beginning to be explored and that deserves future exploration and elucidation [9].

In this context, Weinberger, Brigante, and Nissen’s insights may be helpful [8]. They argue that hypnosis, although seemingly strange is, in fact, a product of normative mentation. This perspective challenges traditional views of hypnosis as a distinct or aberrant state, instead positioning it within the continuum of normal cognitive processes. Their critique of dissociation theories and emphasis on the role of unconscious cognitive processes in hypnosis aligns with the broader theme of an unconscious gatekeeper. By reframing hypnosis as an extension of everyday mental functions, their work contributes to a more nuanced understanding of the interplay between conscious and unconscious processes, further demystifying the nature of hypnosis and placing it squarely within the purview of cognitive science.

Future theoretical work in the field of hypnosis will hopefully continue to explore the dynamic interplay between conscious and unconscious processes during hypnotic states. This might include investigating the mechanisms behind the unconscious “gatekeeper” role, how it modulates information flow between conscious and unconscious realms, and its impact on hypnotic suggestibility and response. Additionally, examining the neurocognitive underpinnings of hypnosis, particularly in the context of theories like massive modularity, connectionism, and neural reuse, could yield insights into how specific brain networks and circuits are involved in and contribute to hypnotic experiences. There also exists a need for empirical studies to validate and refine existing theories, for example, through MRI scans. This research will not only deepen our understanding of hypnosis, but also contribute to the broader discourse on the nature of and interplay between conscious and unconscious processes.
